# Warming neutralizes host-specific competitive advantages between a native and invasive herbivore

**DOI:** 10.1038/s41598-018-29517-0

**Published:** 2018-07-24

**Authors:** Zheng-Hong Lin, Chung-Huey Wu, Chuan-Kai Ho

**Affiliations:** 1 Institute of Ecology and Evolutionary Biology, National Taiwan University, Taipei, Taiwan; 2 Australian Research Council Centre of Excellence for Environmental Decisions, University of Melbourne, Melbourne, Australia; 3 Department of Life Science, National Taiwan University, Taipei, Taiwan

## Abstract

Although native-invasive species interactions have become a common mechanism shaping ecosystems, whether these interactions shift under warming remains unclear. To investigate how warming may affect native and invasive species separately and together (intraspecific and interspecific competition, respectively) and whether any warming impact is resource dependent, we examined the performance of two competing herbivores (native *Pieris canidia* and invasive *P*. *rapae*) on two common host plants under three temperature settings (control, 3 °C, and 6 °C warming using environmental chambers). The results revealed that warming benefited the development and growth of both *Pieris* under intraspecific competition, but the benefits were host-plant dependent. Notably, the native or invasive *Pieris* gained an advantage from interspecific competition (host-plant dependent), but warming neutralized the competitive advantages of either *Pieris* species. These findings raise the possibility that warming-induced shifts in competitive status may become a crucial mechanism shaping ecosystems worldwide, because most ecosystems are challenged by species invasion and warming. Moreover, this study revealed a discrepancy in species thermal performance between intra- and interspecific competition. Therefore, to predict native-invasive species competition under warming, current thermal performance applications should use species performance curves derived from interspecific rather than intraspecific competition studies (although the latter is more readily available).

## Introduction

Species invasion, a worldwide phenomenon, can threaten or alter biodiversity, community structure and ecosystem functions in invaded habitats^1–5^. One of the underlying mechanisms for invasive species’ establishment and impact involves competition between invasive and native species, which has been highlighted in several leading hypotheses in invasion ecology. For example, invasive species can outcompete native species by releasing novel chemical weapons^6,7^, evolving to a level of competitive superiority^8^, but see^9^, possessing a superior adaptation ability^7,10^, or carrying an advantageous life history traits^7,11,12^.

Although studies have explicitly demonstrated how interspecific competition leads to the establishment of invasive species, how this outcome might shift under subsequent environmental changes is unclear. For example, will climate warming (e.g., a higher average temperature or a hotter summer) increase or decrease the competitive advantage of invasive species against native ones? More worryingly, will invasive species in invaded habitats become more invasive under warming? While previous studies have investigated the interplay between climate warming and species invasion, the aforementioned questions remain to be answered. For example, studies have suggested that warming may benefit invasive species by lifting the thermal constraints of their new habitats (e.g., higher latitude)^3,13,14^. Although such studies are invaluable in predicting how warming may facilitate the colonization of new habitats by invasive species, they have offered few hints regarding how warming may affect the current competition between invasive and native species in already invaded habitats.

Other studies based on species’ performance over temperature gradients (i.e., thermal performance) have suggested that warming may benefit invasive species because their thermal tolerance is higher than that of native species (e.g., the “tolerant invaders hypothesis”)^15,16^ (Supplement 1). However, the use of this thermal performance concept (the tolerant invaders hypothesis) to predict species competition outcomes relies on the following assumptions that remain to be tested. (a) This thermal performance concept assumes that native species suffer more (or benefit less) than do invasive ones from warming. However, this assumption does not account for cases where native species can benefit from modest warming as much as invasive ones (e.g., through faster growth and development). These cases may be common; meta-analysis results have indicated that native and non-native species generally respond to warming to a similar degree, although non-native aquatic species may respond more positively to warming than native aquatic ones^17^, but see^18^. (b) The thermal performance concept does not address the effects of resources on the competitive outcomes of native vs. invasive species. It is unclear whether we can assume the same competitive outcome across resource types (e.g., invasive herbivores uniformly outcompete native ones on different host plant species under warming). To advance the application of the thermal performance concept, we should verify whether the competitive outcome is resource (e.g., host plant) dependent, and whether the degree of this resource dependency is mediated by temperature (warming). (c) The thermal performance concept predicts the consequences of warming on interspecific competition based on the thermal performance curves of native and invasive species (Supplement 1). Although not explicitly defined, these curves are most likely derived from experiments *without* interspecific competition settings, because almost all thermal performance data (curves) to date have been generated by studies with intraspecific- or no-competition settings^19–24^. Considering data availability, we need to determine whether we can use thermal performance curves from intraspecific- or no-competition studies (readily available) to predict interspecific competition outcomes under warming (e.g., species composition change) (Supplement 1). This prediction is valid, for example, if the thermal performance curves from intraspecific competition are equal to those from interspecific competition.

Whereas previous studies have proposed that warming may favor invasive species in a native-invasive species competition, we posited that the competition outcome depends on at least three factors. (1) “Hotter is faster”: Individual organisms commonly grow faster under modest warming conditions^25–27^. Therefore, competing native and invasive species will likely both undergo growth acceleration under warming. (2) Degree of growth acceleration: If the growth acceleration under warming is symmetric between native and invasive species (i.e., the species exhibit the same degree of growth acceleration under warming), warming may not change the original competition outcome (e.g., invasive species maintain similar competitive advantages under both control and warming conditions). However, if growth acceleration under warming is asymmetric between the species, then warming will likely alter the original competition outcomes. For example, warming may reinforce, neutralize, or reverse the current superiority of invasive species over native species. (3) Resource dependence: When native and invasive species compete for the same resources, one species may develop more effective adaptations to certain resources than the other species, leading to resource-dependent competition outcomes. To predict warming impact on native-invasive species competition, we should clarify whether the impact is resource-dependent.

**Figure 1:**
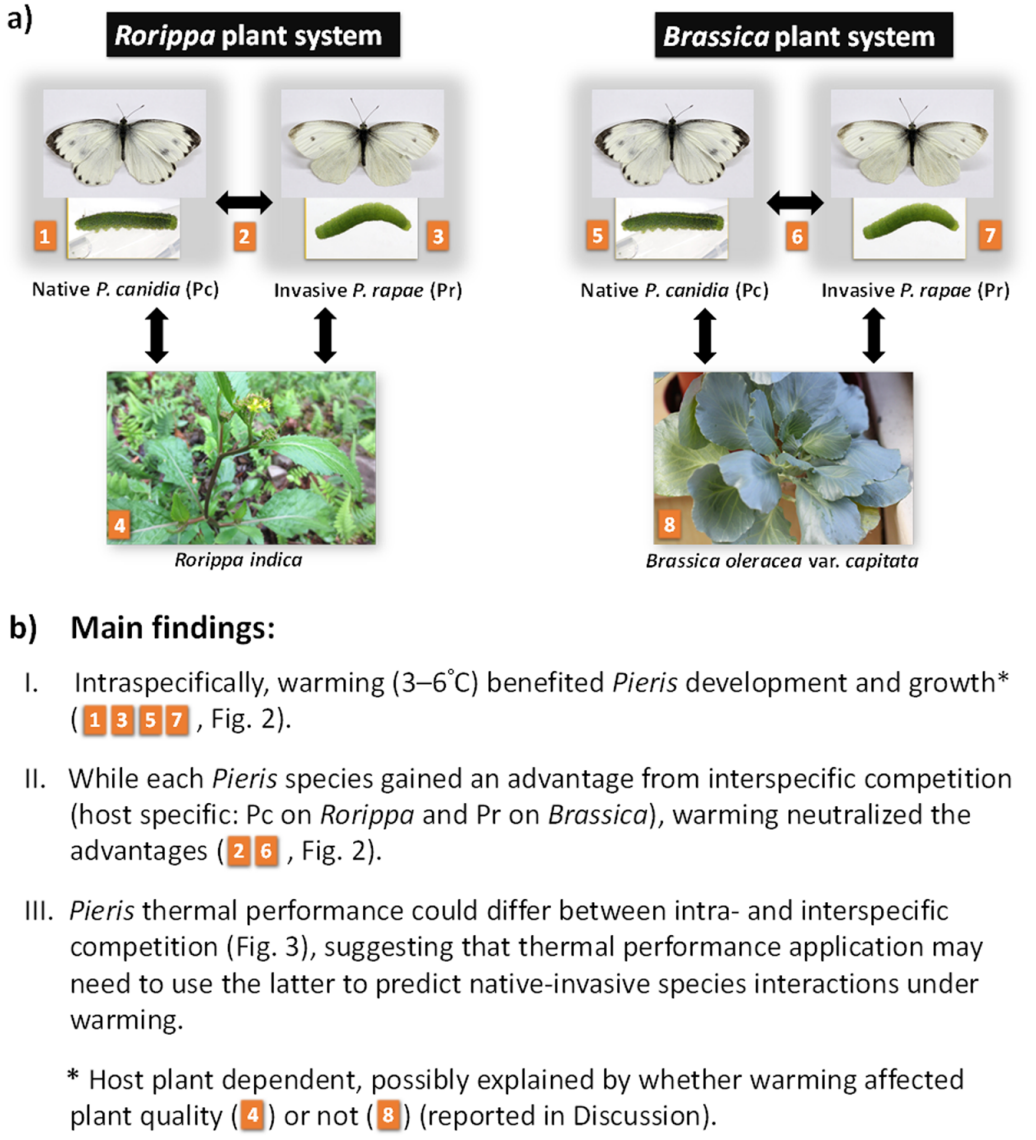
Concept map of this study, comprising its (**a**) systems and (**b**) main findings. To understand the warming impact (+3 °C or +6 °C) on native *Pieris canidia*, invasive *P*. *rapae*, and their competition on major host plants, this study examined the performance of each *Pieris* species under intraspecific competition (1 and 5 for *P*. *canidia*; 3 and 7 for *P*. *rapae*) or interspecific competition (2 and 6) on *Rorippa indica* (4) and *Brassica oleracea* var. *capitata* (8). The horizontal and vertical arrows indicate competition and herbivory, respectively.

To improve our understanding of warming impact on native-invasive species interactions, as well as examining the aforementioned thermal performance concept, we investigated (1) how warming may affect the performance of native or invasive herbivores alone (intraspecific competition) and their current interactions (interspecific competition); (2) whether this warming impact, if it exists, is resource (host plant) dependent; and (3) whether the application of the current thermal performance concept can predict the interspecific competition outcomes of native vs. invasive species through species performance curves derived from intraspecific competition across temperature gradients. Specifically, we experimentally examined the temperature effect (control, +3 °C, +6 °C) on two competing butterfly species in Taiwan (the native *Pieris canidia* and the invasive *P*. *rapae*) on two of their major host plants - variableleaf yellowcress (*Rorippa indica*) and cabbage (*Brassica oleracea* var. *capitata*) (Fig. 1). Temperature was manipulated using environmental chambers. The control temperature (18.5 °C) reflected the average monthly temperature of *Pieris* peak season (details in Methods). The warming conditions (+3 or +6 °C) were comparable to the Intergovernmental Panel on Climate Change (IPCC) prediction for the year 2100^28,29^. *P*. *canidia and P*. *rapae* were among the most common local butterflies, with *R*. *indica* and *B*. *oleracea* as their major host plants in natural and agricultural systems, respectively. We measured *Pieris* performance to demonstrate the warming impact on the native *Pieris*, invasive *Pieris*, and their competition on host plants. Each host plant species system (plant + *Pieris*) was separately examined to enable us to test whether any warming-induced shifts in *Pieris* competition were host plant dependent. Furthermore, we compared the thermal performance of the native and invasive *Pieris* under intraspecific and interspecific competition, in order to examine whether the current thermal performance concept can apply intraspecific data to predict interspecific competition outcomes between native and invasive species.

## Results

### Warming impact on each *Pieris* (intraspecific competition)

**Figure 2:**
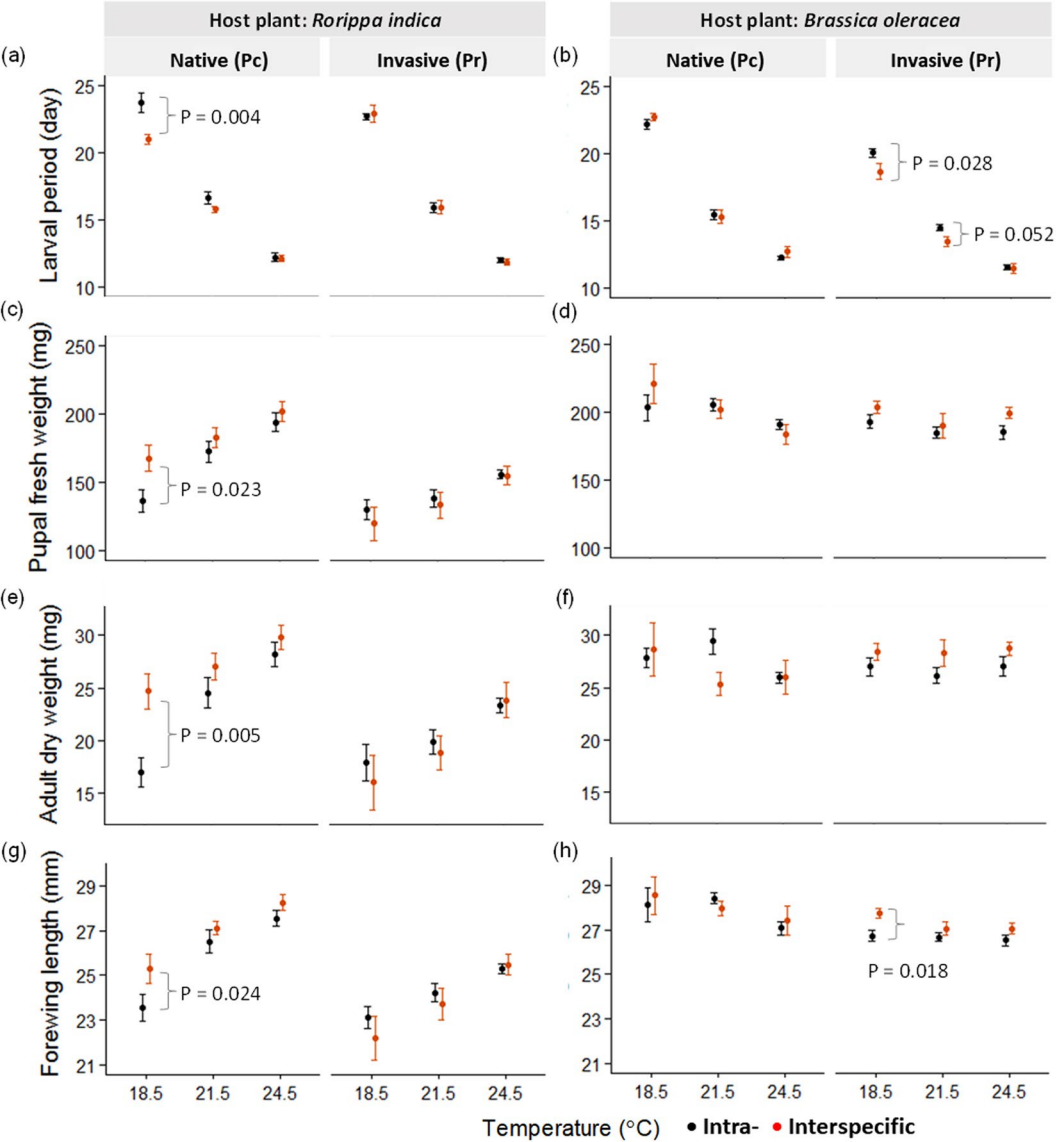
Temperature effect on the performance of two *Pieris* butterfly species under intraspecific (black dots) and interspecific (red dots) competition on two host plant species (*Rorippa indica* and *Brassica oleracea*). *Pieris* performance traits (mean ± SE) include larval period (**a**,**b**), pupal fresh weight (**c**,**d**), adult dry weight (**e**,**f**), and forewing length (**g**,**h**). Pc and Pr indicate the native *Pieris canidia* and invasive *P*. *rapae*, respectively. The temperatures at 18.5 °C, 21.5 °C, and 24.5 °C represent control, 3 °C, and 6 °C warming, respectively. The statistical results are listed in Supplement 5 (ANOVA results), 6 (post hoc comparisons across temperature), and 7 (comparisons between intra- and interspecific competition). The *P*-values in the figure indicate that *Pieris* thermal performance differs between intraspecific and interspecific competition at a specific temperature.

When each *Pieris* species was raised alone (intraspecific competition), warming generally accelerated *Pieris* development (larval period), but the warming impact on *Pieris* growth (body weight and size) was host plant dependent. In the *Rorippa* plant system, warming increased the performance of both *Pieris* in development (e.g., shorter larval period) and growth (e.g., larger pupal fresh weight, larger adult dry weight, longer forewing length) (black dots in Fig. 2a,c,e,g). In contrast to these results, in the *Brassica* plant system, warming increased the developmental performance of both *Pieris* (e.g., shorter larval period), but had minimal effect on the growth performance of *P*. *canidia* (e.g., slightly lower pupal fresh weight under warming) (black dots in Fig. 2b,d,f,h). Detailed statistical results are shown in Supplement 5 (ANOVA results) and 6 (post hoc comparisons across temperature).

### Warming impact on *Pieris* competitive status (intra- vs. interspecific competition)

Although the interspecific competition results were host plant specific (i.e., *P*. *canidia* and *P*. *rapae* had an advantage on *Rorippa* and *Brassica* plants, respectively), warming could shift the competitive status of the native and invasive *Pieris* on each of the two host plant species. Specifically, warming reduced or removed the current advantage of either *Pieris* species. In the *Rorippa* plant system, *P*. *canidia* gained an advantage from interspecific competition under control temperature (18.5 °C), suggested by the higher performance in development (shorter larval period) and growth (larger pupal weight, larger adult dry weight, and longer forewing length) under the interspecific than intraspecific competition treatment (Fig. 2a,c,e,g; orange vs. black dots at 18.5 °C; *P*-values in the figure). However, this competitive advantage of *P*. *canidia* disappeared under 3 °C or 6 °C warming (Fig. 2a,c,e,g), indicating that warming shifted the competitive status of the two *Pieris* on *Rorippa* plants. Detailed statistical results are shown in Supplement 7 (comparisons between intra- and interspecific competitions).

In the *Brassica* plant system, *P*. *rapae* gained an advantage from interspecific competition under control temperature (18.5 °C), suggested by the higher performance in development (shorter larval period) and growth (longer forewing length) under the interspecific than intraspecific competition treatment (Fig. 2b,h; orange vs. black dots at 18.5 °C; *P*-values in the figure). However, this competitive advantage of *P*. *rapae* decreased or disappeared under warming (i.e., larval period (Fig. 2b) and forewing length (Fig. 2h), respectively). This indicates that warming shifted the competitive status of these two *Pieris* on *Brassica* plants, consistent with the results on *Rorippa* plants. Detailed statistical results are shown in Supplement 7.

### Thermal performance concept (intra- vs. interspecific competition)

**Figure 3:**
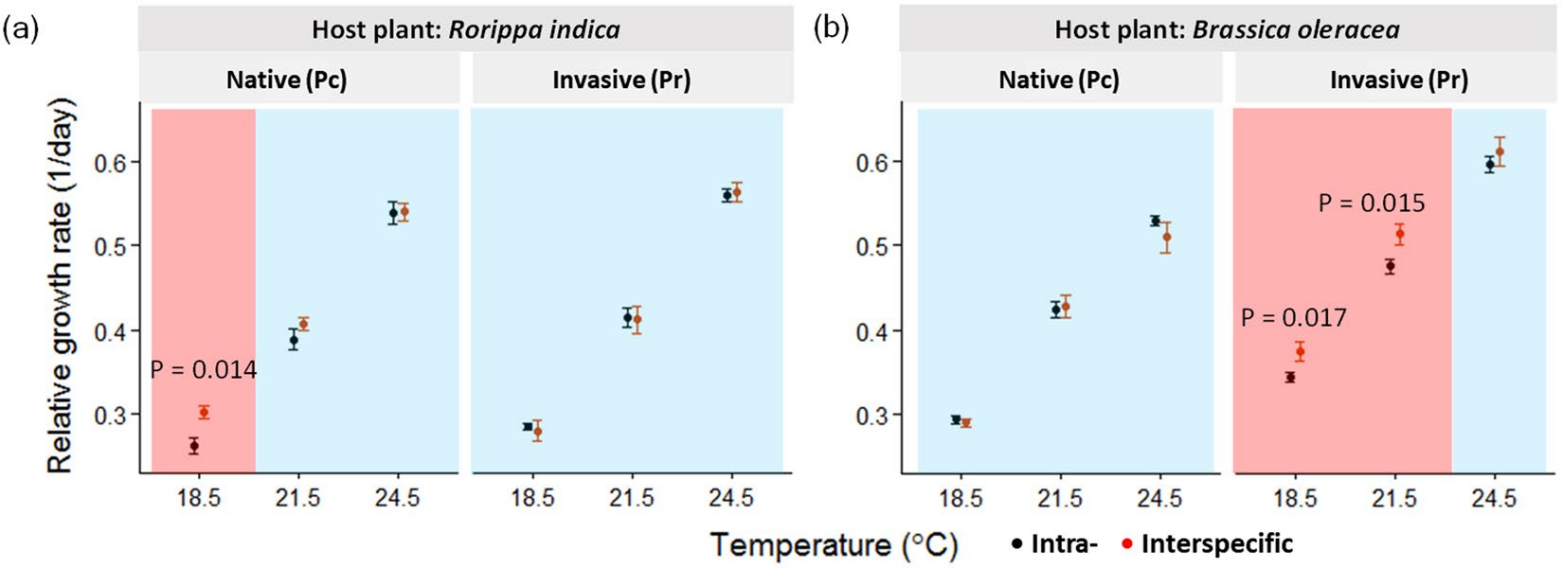
Standardized thermal performance reflected by the relative growth rates (RGRs) of the two *Pieris* butterfly species under intraspecific (black dots) and interspecific (red dots) competition on the two host plant species. Pc and Pr indicate the native *P*. *canidia* and invasive *P*. *rapae*, respectively. The temperatures at 18.5 °C, 21.5 °C, and 24.5 °C represent control, 3 °C, and 6 °C warming, respectively. Panels (**a**,**b**) show *Pieris* RGRs (mean ± SE) on *R*. *indica* and *B*. *oleracea* plants, respectively. Color highlights indicate difference (red; *P*-value provided) or similarity (blue) in the *Pieris* thermal performance (RGR) between intraspecific and interspecific competition.

This study standardized the thermal performance of *Pieris* by calculating their relative growth rates (RGRs). The RGR results for both *Pieris* showed that the thermal performance under intraspecific competition could be different from that under interspecific competition. In other words, the results demonstrate a potential pitfall in applying the thermal performance concept, namely that using thermal performance curves derived from intraspecific competition settings (readily available for some species) may not predict the real outcome of interspecific competition under warming. Specifically, in the *Rorippa* plant system, *P*. *canidia* performance under intraspecific competition was lower than that under interspecific competition at 18.5 °C (Fig. 3a). *P*. *rapae* did not exhibit this intra- vs. interspecific discrepancy (Fig. 3a). However, in the *Brassica* plant system, *P*. *rapae* performance under intraspecific competition was lower than that under interspecific competition at 18.5 °C and 21.5 °C (Fig. 3b). *P*. *canidia* did not exhibit this intra- vs. interspecific discrepancy (Fig. 3b). Detailed statistical results are provided in Supplement 7.

## Discussion

Because of the ubiquity of invasive species, competition between native and invasive species has become a new norm of species interactions in natural and agricultural communities. Although previous studies have demonstrated the effects of this new norm on biodiversity, community structure and ecosystem functions, further studies are required to understand how these effects may shift under environmental changes (e.g., a hotter summer or warmer average temperature). This study examined the warming impact on current competition of native and invasive herbivores (two *Pieris* butterfly species) on two host plant species and obtained the following main findings (Fig. 1). (1) Intraspecifically, warming accelerated *Pieris* development (larval period), but the warming impact on *Pieris* growth (body weight and size) was host plant dependent. (2) Although each *Pieris* species might gain an advantage from interspecific competition (host plant specific: native *P*. *canidia* on *Rorippa* and invasive *P*. *rapae* on *Brassica*), warming neutralized the advantages. In other words, warming did not necessarily benefit invasive species as predicted by the current thermal performance concept (the tolerant invaders hypothesis). (3) The thermal performance of a species under intraspecific competition could differ from that under interspecific competition. Therefore, to more accurately predict native-invasive species interactions and the outcomes under warming (e.g., species composition change), the application of the current thermal performance concept may need to use species performance curves derived from interspecific rather than intraspecific competition studies (although the latter can be readily available). Based on the aforementioned main findings, we discuss below why warming can be good or bad news for native species, how warming could neutralize the current advantage of native or invasive species, why the warming impact on herbivore growth is host plant dependent, and how to improve the application of the thermal performance concept to more accurately predict native-invasive species competition.

### Warming impact on native-invasive species competition

Whether invasive species in invaded habitats will become more invasive against native species under warming is a major concern for ecologists and conservation practitioners. This concern, however, is not warranted in this study. When the invasive or native *Pieris* gained an advantage from the interspecific competition on a host plant species, warming by 3–6 °C neutralized this advantage (Figs 2 and 3). When neither the invasive nor native *Pieris* gained an advantage from interspecific competition, warming by 3–6 °C did not change the pattern. In summary, warming seemed to homogenize the performance of the native and invasive species in this study. If this proves to be a common pattern, modest warming (e.g., by 3–6 °C) can be expected to bring good and bad news for invaded habitats. The good news is that in habitats where invasive species currently dominate, native species populations may have a chance to bounce back under modest warming. The bad news is that in habitats where native species currently dominate, exotic species may become a greater threat under modest warming.

How did warming neutralize the advantage for the invasive and native *Pieris* in an interspecific competition? This neutralization may be a result of “hotter is faster” and the asymmetric growth acceleration of each species under warming. First, the concept of “hotter is faster” is a common pattern in ectotherms – individuals grow faster under modest warming conditions^25–27^. Faster growth can lead to advantages in resource competition, such as in cases of asymmetric competition^30–33^. In our study, warming induced faster growth in both *Pieris* (Figs 2a,b and 3) and possibly made both *Pieris* equally effective at competing for food resources. Consequently, this warming effect may outweigh and mask the difference in competitive ability between the two *Pieris* that was originally revealed at the control temperature. Second, asymmetric growth acceleration between the two *Pieris* may lead to warming-induced neutralization (e.g., higher growth acceleration in the less competitive species under warming). A supporting example came from the *Rorippa* plant system, where *P*. *canidia*, but not *P*. *rapae*, gained an advantage from interspecific competition at the control temperature (red highlight in Fig. 3a). However, 6 °C warming accelerated the growth of *P*. *canidia* by 79% (RGR 0.302 vs. 0.540) but that of *P*. *rapae* by 102% (RGR 0.262 vs. 0.530). The higher growth acceleration in *P*. *rapae* over *P*. *canidia* under warming (permutation test, *P* = 0.033) may have been enough to offset the original competitive advantage of *P*. *canidia* (at control temperature), leading to the observed neutralization in interspecific competition.

A further question is why the invasive and native *Pieris* gained an advantage (host plant dependent) in an interspecific competition. Although this study was not designed to examine the underlying mechanisms, we speculate that one species may adapt slightly better to a host plant than the other species, such as an ability to precisely locate more nutritious plant tissue on a single leaf or plant. Therefore, this species could gain an advantage under interspecific competition. We did not observe noticeable food partitioning between the two species or behavioral changes when the two species were put together. Therefore, food partitioning and behavioral shifts are probably not strong candidates for the underlying mechanisms.

### Warming impact is host plant dependent

The warming impact on *Pieris* growth (body weight and size) was host plant dependent regardless of competition type, which highlights the need to consider the host plant (resource) effect in predicting the competition results of native vs. invasive herbivores under climate change. For example, warming generally increased *Pieris* growth performance on *Rorippa* plants but not on *Brassica* plants under intra- and interspecific competition (Fig. 2c vs. 2d,e vs. 2g vs. 2h; Supplement 6). Why was warming impact on *Pieris* competition host plant dependent? Although this question is outside the scope of this study, we suspect that the dependence is partially due to a different response of plant quality to warming. For example, warming increased leaf N content in *Rorippa* plants (1.70 ± 0.11%, 2.36 ± 0.17%, and 3.74 ± 0.17% for control, +3 °C, and + 6 °C, respectively [mean ± SE]; *P* < 0.0001) but not in *Brassica* plants (5.46 ± 0.08%, 5.38 ± 0.15%, and 5.50 ± 0.06% for control, +3 °C, and +6 °C, respectively [mean ± SE]; *P* = 0.77). Considering that higher plant N content usually indicates higher food quality for herbivores^34–37^, it is reasonable that we observed a better *Pieris* growth under warming on *Rorippa* (increasing N%) but not *Brassica* plants (no change in N%). Because *Rorippa* and *Brassica* are major host plants for *Pieris* in natural and agricultural systems, respectively, our results call for research to investigate whether the warming impact on native and invasive herbivores commonly differs between natural and agricultural systems – for example, because of systematic differences in plant N% and plant response to warming.

### Improving thermal performance application to predict native-invasive species competition

We suggest that the application of the thermal performance concept should use species performance curves derived from interspecific competition to more accurately predict the competition outcome between native and invasive species populations in invaded habitats. Ecologists and conservation practitioners increasingly apply the thermal performance concept to predict such outcomes under climate change (e.g., species composition change). Although not specified, these predictions are most likely based on species thermal performance derived from intraspecific- or no-competition, rather than interspecific-competition, settings because of data availability. However, our study highlights the risk of this practice, because a native or invasive species could perform more favorably under interspecific rather than intraspecific competition (*P*. *canidia* on *Rorippa* at 18.5 °C in Fig. 3a; *P*. *rapae* on *Brassica* at 18.5 °C and 21.5 °C in Fig. 3b). In other words, a prediction based on intraspecific competition results may underestimate the performance of native or invasive species under real interspecific competition.

While our study highlights an existing discrepancy between species performance under intra- and interspecific competition, this discrepancy can be temperature dependent – it may decrease or disappear under warming (Fig. 3a,b). Does this mean that the performance of native and invasive species will converge under climate warming? Does this also mean that the abiotic factor (temperature) will overpower biotic factors (e.g., interspecific competition and host plant quality) under warming? Future studies of different systems could verify the generalizability of our results and help advance climate change ecology.

## Conclusions

As natural and agricultural systems increasingly face double jeopardy, namely species invasion and climate warming, this study demonstrated how competition between native and invasive species may proceed under climate warming (with potential caveats in Supplement 8). The major findings suggest the following points. (1) Intraspecifically, modest warming (3–6 °C) could benefit the development and growth of native or invasive species, potentially in a resource-dependent manner. (2) Warming did not necessarily benefit invasive species as predicted by the current thermal performance concept (the tolerant invaders hypothesis). Instead, native or invasive species may gain an advantage from interspecific competition (resource dependent), and modest warming (3–6 °C) can neutralize the competitive advantages of either species. This warming-induced shift in competitive status may serve as a vital mechanism shaping natural or agricultural systems worldwide, because most ecosystems already contain invasive species and face climate warming. (3) To more accurately predict the competitive outcomes of native vs. invasive species under climate warming (e.g., species composition change), the application of the thermal performance concept should use species performance curves derived from interspecific rather than intraspecific competition studies.

## Methods

### Species

#### *Pieris canidia* and *P. rapae* butterflies

*P*. *canidia* lives in East Asia, Southeast Asia and South Asia^38^. *P*. *rapae*, originally from Eurasia, has been introduced to North America, Australia, New Zealand, Japan and Taiwan^39–42^. In Taiwan, the native *P*. *canidia* and invasive *P*. *rapae* are common from late autumn to spring in lowland areas, and their larvae overlap in host plants (wild and agricultural Brassicaceae plants). The overlap in their peak season and host plants suggests an interspecific competition in the field, supported by personal observations that the *P*. *canidia* population has decreased since the introduction of *P*. *rapae*^40,43^. In addition, their larvae have been recorded on adjacent or the same individual plants in the field (Lin and Ho, *personal observations*). The detailed methods of *Pieris* collection used in this study are listed in Supplement 3.

#### *Rorippa indica* and *Brassica oleracea* var. *capitata*

In Taiwan, *R*. *indica* (variableleaf yellowcress) and *B*. *oleracea* var. *capitata* (cabbage), major wild and agricultural Brassicaceae plant species, respectively, are among the most common host plants of *Pieris* (Lin and Ho, *personal observations*), and thus were selected for this study. Both plants contain chemicals for protection against herbivores (e.g., glucosinolates)^44^, although the *Pieris* species in this study are considered as specialists of the Brassicaceae plants. The detailed methods of plant collection are listed in Supplement 3.

### Warming impact on *Pieris* competition

To test the warming impact on the native and invasive *Pieris* alone and together (intra- and interspecific competition, respectively), we raised *Pieris* on host plants in laboratory experiments with a factorial design crossing temperature and competition treatments (Supplement 2). Each host plant system was examined in a separate experiment because of logistical concerns. The temperature treatment maintained species in environmental chambers at 18.5 °C, 21.5 °C, and 24.5 °C, thereby simulating control, 3 °C, and 6 °C warming, respectively. The control temperature reflected the average temperature in March (1981–2010) in the Taipei Basin (Central Weather Bureau 2011), the month when field *Pieris* density started to peak (Lin and Ho *unpublished data*). Warming at 3 °C or 6 °C was set in accordance with the IPCC prediction for the year 2100^25,26^. The competition treatment consisted of the following four groups: (a) 4 *P*. *canidia*, (b) 2 *P*. *canidia* and 2 *P*. *rapae*, (c) 4 *P*. *rapae* larvae on a host plant, and (d) a host plant only. The larval density (n = 4/plant) was within the range of the field density (Lin and Ho *unpublished data*). These treatment combinations (temperature × competition) enabled us to examine the warming impact on intra- and interspecific competition among *Pieris* (group a, b, and c) and on host plant quality (group d). Understanding the response of host plant quality to warming may help explain the underlying mechanisms for warming impact on herbivore performance. Each treatment combination was replicated in 6 cages that initially housed 1^st^ instar *Pieris* and/or plants originally from our 3 sites (i.e., 2 cages per site) (Supplement 2).

To quantify *Pieris* performance, we monitored *Pieris* daily and measured traits related to development (e.g., larval period) and growth (e.g., pupal fresh weight, adult dry weight, and adult forewing length). These traits are generally critical because shorter developmental time may reduce predation risk, and a larger body size is often linked to higher fecundity in arthropods. To standardize the thermal performance of the two *Pieris* species, we calculated their relative growth rates (RGRs) as RGR = (LnW_1_ − LnW_0_)/(T_1_ − T_0_). W_1_ and W_0_ represent the final (pupal weight) and initial (1^st^ instar) weight at times T_1_ and T_0_, respectively. Considering that the 1^st^ instars were very lightweight and vulnerable, we estimated their weight by averaging more than 20 individuals (per species) that would not be used in the laboratory experiments. To explain any host-plant-dependent performance in *Pieris*, we analyzed leaf N content, a plant quality trait generally critical to herbivore performance (results are reported in Discussion). Further measurement details are given in Supplement 4.

### Data analysis

To understand the warming impact on *Pieris* performance, we applied a linear mixed model using the lmer function from the lme4 package in R. For the *Pieris* larval period, pupal fresh weight, adult dry weight, and forewing length, our model included temperature treatment, competition type, and *Pieris* gender as fixed effects, with collection site and cage effect as random intercept effects. Each *Pieris* species was analyzed separately, with individuals as the unit of analysis. Competition type (intra- or interspecific competition) was derived from our competition treatment. Intraspecific competition type included individuals from the competition treatment group a (*P*. *canidia* only) or c (*P*. *rapae* only). Interspecific competition type included individuals from the competition treatment group b (*P*. *canidia* and *P*. *rapae* together). Considering that each host plant system (*Rorippa* or *Brassica* plants and *Pieris*) was examined in a separate experiment, we analyzed each system separately. Pairwise comparisons of *Pieris* performance between intra- and interspecific competition were made using lsmeans with gender pooled, except for RGRs. The comparisons of RGRs (ratio data) were made using permutation tests (5000 iterations) to generate frequency distributions for testing hypotheses (significant level = 0.05).

## Electronic supplementary material

Supplementary information
