# Supplementary material for: Warming neutralizes host-specific competitive advantages between a native and invasive herbivore
Source: Sci Rep. 2018 Jul 24;8:11130. doi: 10.1038/s41598-018-29517-0 (PMC6057923; doi:10.1038/s41598-018-29517-0)
Supplement: Supplementary file 1 — Supplementary information [file 41598_2018_29517_MOESM1_ESM.pdf]

Warming neutralizes host-specific competitive advantages between a native and invasive herbivore

Zheng-Hong Lin[1], Chung-Huey Wu[1,2], Chuan-Kai Ho[1,3*]

[1]Institute of Ecology and Evolutionary Biology, National Taiwan University, Taipei, Taiwan

[2]Australian Research Council Centre of Excellence for Environmental Decisions, University of Melbourne, VIC, Australia

[3]Department of Life Science, National Taiwan University, Taipei, Taiwan

* Correspondence author. Email: ckho@ntu.edu.tw

Supplement 1. Applying thermal performance to predict native-invasive species competition under warming.

Thermal performance

Vital organismal performance (e.g., growth, development, reproduction and locomotion) is strongly affected by temperature[1-4]. The relationship between organismal performance and temperature can be described as thermal performance (curves)[1,5,6]. Given the ability to predict organismal performance under temperature gradients, thermal performance curves have been widely used by biologists in recent years to predict climate change impact on species performance (e.g., fitness), evolution (e.g., adaptation), and interactions (e.g., native-invasive species competition)[2,7,8].

Native-invasive species competition under warming

The figure below depicts conceptual thermal performance curves of native and invasive species under intraspecific competition (native invasive) and interspecific competition (native invasive). Thermal performance curves have been used by ecologists to predict climate warming impact on native vs. invasive species; for example, warming may benefit invasive species because of their higher thermal tolerance (e.g., > ; the tolerant invaders hypothesis). The application of this concept tends to use thermal performance curves derived from intraspecific- or no-competition settings due to data availability. However, this approach may underestimate the effect of interspecific competition (native vs. invasive species) and fail to predict the real outcome. For example, this approach may predict a similar competitive performance in native (N) and invasive (I) species at temperature X °C ($N_1 \approx I_1$; vs.). However, this prediction may not reflect the real competitive outcome ($N_2 > I_2$; vs.). This discrepancy (intra vs. inter) in species performance curves is also demonstrated in the inset (red highlight). Whether this discrepancy and competitive outcomes are resource dependent remains to be determined (e.g., host plant specific for native and invasive herbivores).

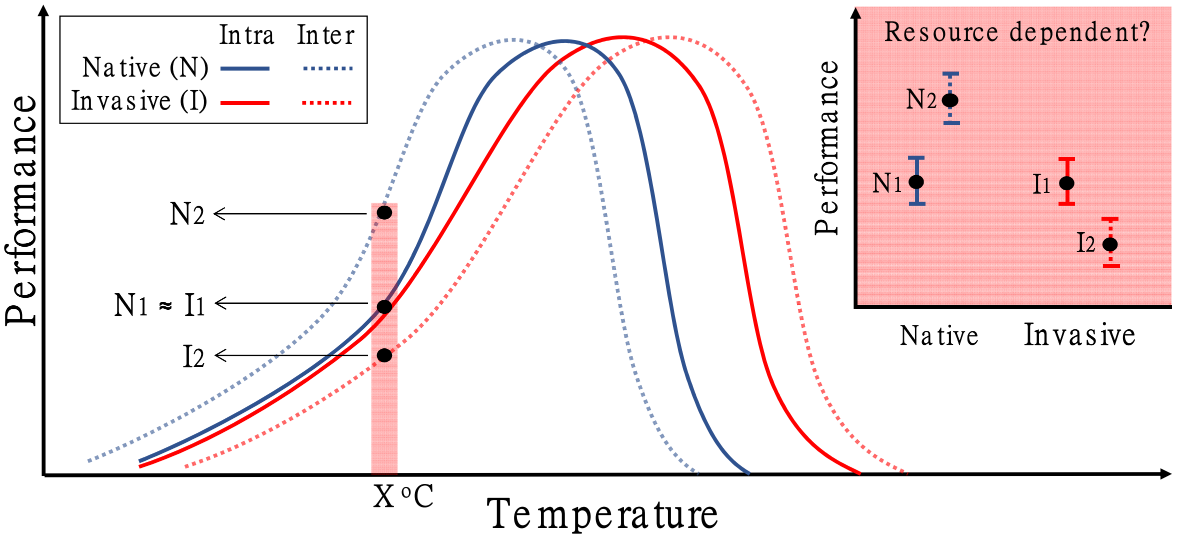

Literature cited for Supplement 1

1 Huey, R. B. & Stevenson, R. D. Integrating thermal physiology and ecology of ectotherms - discussion of approaches. *Am Zool* **19**, 357-366 (1979).

2 Deutsch, C. A. *et al.* Impacts of climate warming on terrestrial ectotherms across latitude. *P Natl Acad Sci USA* **105**, 6668-6672 (2008).

3 Stevenson, R. D., Peterson, C. R. & Tsuji, J. S. The thermal-dependence of locomotion, tongue flicking, digestion, and oxygen-consumption in the wandering garter snake. *Physiol Zool* **58**, 46-57 (1985).

4 Wang, Y. J., Nakazawa, T. & Ho, C. K. Warming impact on herbivore population composition affects top-down control by predators. *Sci Rep-Uk* **7**, 941 (2017).

5 Jonsson, B., Forseth, T., Jensen, A. J. & Naesje, T. F. Thermal performance of juvenile Atlantic Salmon, *Salmo salar* L. *Funct Ecol* **15**, 701-711 (2001).

6 Sinclair, B. J. *et al.* Can we predict ectotherm responses to climate change using thermal performance curves and body temperatures? *Ecol Lett* **19**, 1372-1385 (2016).

7 Cheng, B. S., Komoroske, L. M. & Grosholz, E. D. Trophic sensitivity of invasive predator and native prey interactions: integrating environmental context and climate change. *Funct Ecol* **31**, 642-652 (2016).

8 Kingsolver, J. G. & Buckley, L. B. Quantifying thermal extremes and biological variation to predict evolutionary responses to changing climate. *Philos T R Soc B* **372** (2017).

Supplement 2. Detailed experimental design.

To test the warming impact on the intra- and interspecific competition of *Pieris*, we raised *Pieris* on each host plant species in a factorial experiment crossing temperature and competition treatments (Fig. S2-1 and S2-2). The temperature treatment involved maintaining *Pieris* and host plants in environmental chambers at 18.5 ºC, 21.5 ºC, and 24.5 ºC (control, 3 ºC, and 6 ºC warming, respectively). The chambers were set at 70% relative humidity (RH) on a 12-hr light: 12-hr dark cycle, approximately similar to the condition in *Pieris* peak season (March–April) in the Taipei area. The competition treatment included a) 4 *P. canidia,* b) 2 *P. canidia* and 2 *P. rapae,* c) 4 *P. rapae* larvae on a host plant, and d) a host plant only. This enabled us to examine intraspecific competition (a, c), interspecific competition (b), and plant performance (d). To conduct the competition treatment, we added the first instar larvae of *Pieris canidia* (Pc) and/or *P. rapae* (Pr) to a caged host plant (*R. indica* or *B. oleracea* var. *capitata*). Each cage (20 cm [diameter] × 75 cm [height]) was made of transparent nylon mesh (400 holes/cm^2). This cage system enabled *Pieris* to grow normally and reach adulthood in our previous studies.

Figure S2-1. Experimental design of this study, which crossed temperature (18.5 ºC, 21.5 ºC, 24.5 ºC) and competition treatments. The competition treatment included a) 4 *P. canidia* (Pc), b) 2 *P. canidia* and 2 *P. rapae* (Pc + Pr), c) 4 *P. rapae* (Pr) larvae on a host plant, and d) a host plant only. *Pieris* larvae and host plants (*R. indica* or *B. oleracea*) were kept in cages. Details for each treatment combination (e.g., blue rectangle) are demonstrated in Fig. S2-2.

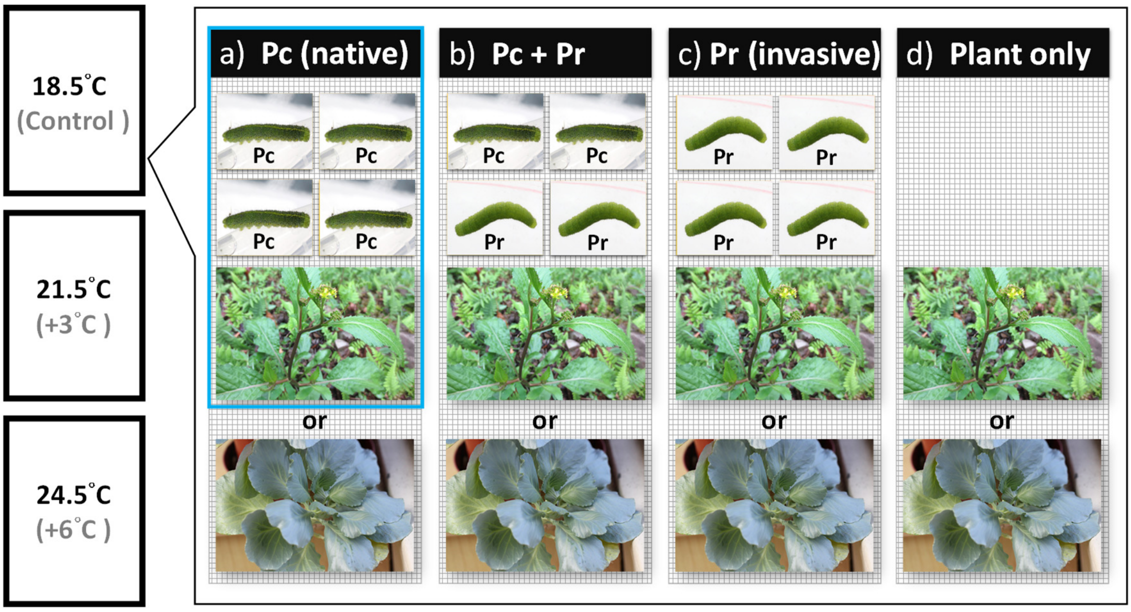

Figure S2-2. Each treatment combination had 6 cages for each host plant system. Each cage initially housed four 1st instar *Pieris* and a plant originally collected from our three study sites: National Taiwan University Horticulture Experimental Farm (N), Tu-Cheng (T), and Song-Shan (S) (site details in Supplement 3).

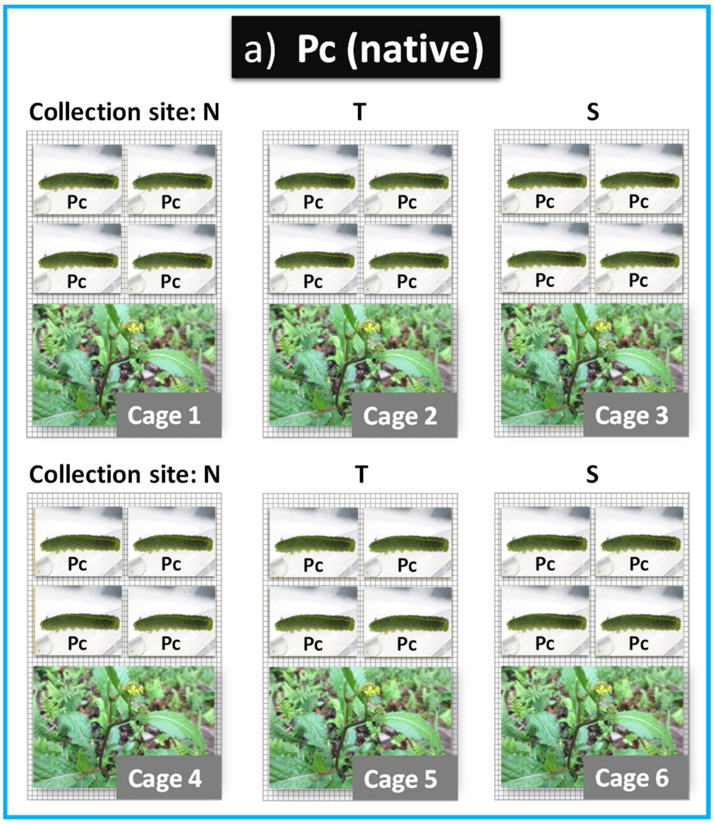

Supplement 3. Species collection methods.

Pieris canidia and P. rapae butterflies

P. canidia and *P. rapae* adults were collected from three sites in the Taipei area to prevent the idiosyncratic effect: National Taiwan University (NTU) Horticulture Experimental Farm (25.010821 N, 121.544493 E), Tu-Cheng (24.968384N, 121.450006E), and Song-Shan (25.029388 N, 121.578491 E). Each site or surrounding area contained the focal host plants of this study (*R. indica* and *B. oleracea* var. *capitata*). After being collected between November and April, *Pieris* (10–12 individuals / sex / species) were kept in mesh cages (80 cm [H] × 50 cm [L] × 50 cm [W]) in the laboratory and allowed to lay eggs on a host plant species to be used in the upcoming experiments (*R. indica* or *B. oleracea*). Once hatched, the first instar caterpillars from the three sites (origins) were used for *Pieris* competition experiments.

Rorippa indica and Brassica oleracea var. capitata

R. indica seeds were harvested from or near the sites where *Pieris* samples were collected and stored at 4°C in a refrigerator until use. The seeds were sown in potting soil in a greenhouse at NTU, and then the seedlings (2-week old) were transferred to pots (15.5 cm [diameter] × 16 cm [height]) in environmental chambers under the temperature settings of this study. When the *Pieris* competition experiments started, the *R. indica* (6-month old) were flowering and fruiting, similar to the condition when *Pieris* became abundant on *R. indica* in the field in winter and spring. The origins (collection sites) of *R. indica* and *Pieris* were paired to simplify the experimental design and prevent potential errors due to local adaptation.

The seeds of *B. oleracea capitata* var. *capitata* cv. Kao-Fong were purchased from Known-You Seed Company. They were sown in potting soil in a greenhouse at NTU, and then the seedlings (1-week old) were transferred to pots (15.5 cm [diameter] × 16 cm [height]) in environmental chambers under the temperature settings of this study. When the *Pieris* competition experiments began, the *B. oleracea* were 6-week old, similar to the condition when *Pieris* started to colonize *B. oleracea* on farm land.

Supplement 4. Species trait measurement.

Pieris traits

Pieris larval were monitored on a daily basis to determine the larval period. Because the fresh pupae were fragile, we measured the pupal weight 1 day after pupation to reduce potential damage. Weight was measured using a digital balance to the nearest 0.0001 g. Pupae from the same cage were then kept in a plastic cup (9 cm [diameter] × 5 cm [height]) with straws and tissue paper (Fig. S4). The straws (numbered) maintained the pupae in their normal upright position and enabled us to identify each individual. After the adults had emerged, they were sexed, euthanized by freezing, and measured for fresh weight and forewing length. Forewing length (from wing joint to wing tip) was measured using a digital caliper to the nearest 0.01 mm. These specimens were then dried in an oven (60 ºC) for 3 days, and their dry weight was measured.

Plant quality trait

Three mature leaves were randomly selected from each individual plant in competition treatment group d (host plant only; see Methods). The leaves were oven dried at 40ºC for one week and then ground to fine powder with a pestle and mortar. Approximately 2 mg of powder from each individual plant was sealed in a tin capsule, and the nitrogen content was analyzed using an elemental analyzer coupled with an isotope ratio mass spectrometer in the Stable Isotope Laboratory at the Institute of Ecology and Evolutionary Biology, National Taiwan University.

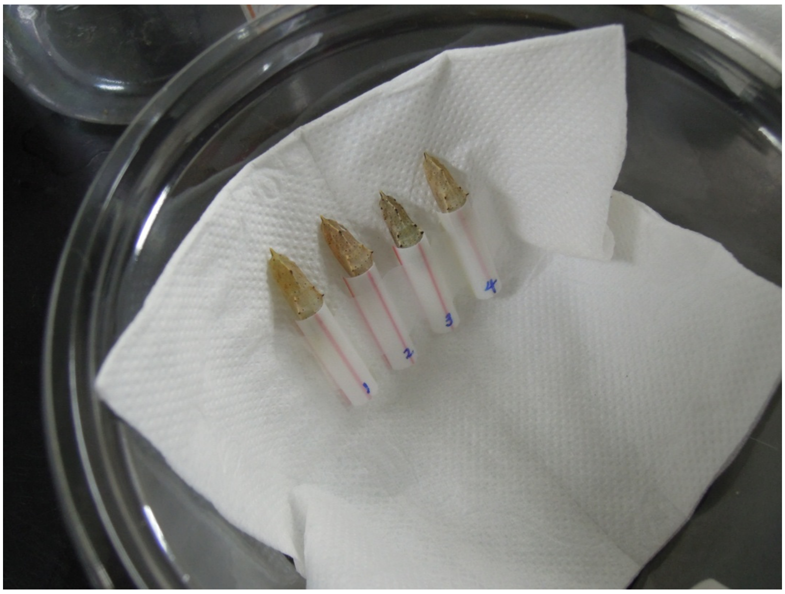

Figure S4. *Pieris* pupae (n=4) from the same cage were stored upright in a plastic container.

Supplement 5. ANOVA table of linear mixed model. CP = competition treatment (intraspecific or interspecific); Temp = temperature treatment (18.5 ºC, 21.5 ºC, and 24.5 ºC, representing control, 3 ºC, and 6 ºC warming, respectively).

Host plant: *Rorippa indica*

Traits	*N*	Fixed effect	*F*	DF	DF.res	*P*
Larval period						
Pc	94	**CP**	4.82	1	28.18	**0.04***
		**Temp**	107.87	2	28.15	**<0.001*** **
		Gender	0.33	1	65.48	0.57
		CP x Temp	2.52	2	28.14	0.10
		CP x Gender	0.00	1	65.80	0.99
		Temp x Gender	1.92	2	65.60	0.15
		CP x Temp x Gender	1.47	2	65.62	0.24
Pr	91	CP	0.01	1	29.62	0.92
		**Temp**	278.40	2	29.32	**<0.001*** **
		Gender	0.21	1	74.20	0.65
		CP x Temp	0.03	2	29.13	0.97
		CP x Gender	0.25	1	73.51	0.62
		Temp x Gender	0.21	2	73.77	0.81
		CP x Temp x Gender	0.72	2	73.75	0.49
Pupal fresh weight						
Pc	94	CP	3.89	1	28.97	0.06
		**Temp**	10.19	2	28.91	**<0.001*** **
		**Gender**	7.16	1	69.43	**<0.01** **
		CP x Temp	1.27	2	28.89	0.19
		CP x Gender	0.11	1	70.94	0.84
		Temp x Gender	0.34	2	70.88	0.90
		CP x Temp x Gender	1.44	2	70.92	0.17

Pr	91	CP	0.13	1	29.36	0.73
		**Temp**	7.72	2	29.04	**<0.01****
		Gender	3.31	1	73.49	0.07
		CP x Temp	0.14	2	28.83	0.87
		CP x Gender	0.24	1	73.27	0.63
		Temp x Gender	1.27	2	72.58	0.29
		CP x Temp x Gender	1.05	2	72.50	0.36
Adult dry weight						
Pc	94	**CP**	7.19	1	29.21	**0.01***
		**Temp**	11.02	2	29.15	**<0.001*****
		Gender	0.00	1	70.55	0.98
		CP x Temp	1.71	2	29.13	0.20
		CP x Gender	0.00	1	71.98	0.97
		Temp x Gender	0.89	2	71.88	0.41
		CP x Temp x Gender	1.68	2	71.91	0.19
Pr	91	CP	0.05	1	29.13	0.82
		**Temp**	7.65	2	28.84	**0.002****
		Gender	0.22	1	72.62	0.64
		CP x Temp	0.07	2	28.64	0.93
		CP x Gender	0.18	1	72.37	0.67
		Temp x Gender	2.17	2	71.79	0.12
		CP x Temp x Gender	1.18	2	71.72	0.31
Forewing length						
Pc	94	**CP**	4.44	1	29.99	**0.04***
		**Temp**	15.52	2	29.90	**<0.001*****
		Gender	5.51	1	72.36	0.02
		CP x Temp	1.00	2	29.88	0.38
		CP x Gender	0.10	1	74.43	0.75

		Temp x Gender	0.62	2	74.17	0.54
		CP x Temp x Gender	0.51	2	74.20	0.61
Pr	90	CP	0.12	1	29.51	0.73
		**Temp**	12.31	2	29.17	**<0.001*** **
		**Gender**	4.12	1	73.85	**<0.05***
		CP x Temp	0.38	2	28.94	0.69
		CP x Gender	1.15	1	73.64	0.29
		Temp x Gender	1.31	2	72.91	0.28
		CP x Temp x Gender	1.07	2	72.82	0.35

Pr: *Pieris rapae*; Pc: *Pieris canidia*

CP: Competition type (intra- vs. interspecific); Temp: Temperature.

N: sample size; *F*: ANOVA F test statistics; DF: degree of freedom; DF.res: residual degree of freedom, estimated using Kenward-Roger method; *P*: p-value, with significant terms in bold. * *P*<0.05; ** *P*<0.01; *** *P*<0.001

Host plant: *Brassica oleracea*

**Traits**	***N***	**Fixed effect**	***F***	**DF**	**DF.res**	***P***
Larval period						
Pc	51	CP	0.01	1	22.56	0.91
		**Temp**	336.97	2	21.42	**<0.001*** **
		Gender	1.49	1	35.07	0.23
		CP x Temp	1.37	2	20.99	0.27
		CP x Gender	0.30	1	35.02	0.59
		Temp x Gender	3.18	2	34.70	0.05
		CP x Temp x Gender	0.43	2	34.65	0.65
Pr	107	**CP**	7.50	1	32.85	**<0.01*** *
		**Temp**	204.78	2	32.80	**<0.001*** **
		Gender	0.00	1	87.59	0.97
		CP x Temp	1.06	2	32.75	0.36
		CP x Gender	0.94	1	88.81	0.33

		Temp x Gender	0.23	2	88.10	0.80
		CP x Temp x Gender	0.09	2	87.97	0.92
Pupal fresh weight						
Pc	51	CP	0.23	1	24.45	0.64
		**Temp**	5.96	2	23.47	**<0.01****
		Gender	1.27	1	36.36	0.27
		CP x Temp	2.58	2	22.88	0.10
		CP x Gender	0.64	1	36.56	0.43
		Temp x Gender	1.63	2	36.02	0.21
		CP x Temp x Gender	0.69	2	36.36	0.51
Pr	107	**CP**	3.69	1	34.70	0.06
		Temp	1.23	2	34.65	0.31
		Gender	1.10	1	89.91	0.30
		CP x Temp	0.25	2	34.57	0.78
		CP x Gender	0.18	1	90.56	0.67
		Temp x Gender	0.50	2	89.81	0.61
		CP x Temp x Gender	0.13	2	89.91	0.88
Adult dry weight						
Pc	51	CP	0.00	1	21.98	0.95
		Temp	2.82	2	20.68	0.08
		Gender	1.94	1	32.80	0.17
		CP x Temp	0.82	2	20.30	0.46
		CP x Gender	1.14	1	32.68	0.29
		Temp x Gender	2.96	2	32.34	0.07
		CP x Temp x Gender	0.73	2	32.34	0.49
Pr	107	CP	3.32	1	33.79	0.08
		Temp	0.11	2	33.74	0.89

		Gender	2.94	1	88.97	0.09
		CP x Temp	0.02	2	33.68	0.98
		CP x Gender	0.05	1	89.84	0.83
		Temp x Gender	0.41	2	89.10	0.67
		CP x Temp x Gender	0.32	2	89.11	0.73
Forewing length						
Pc	47	CP	0.43	1	23.56	0.52
		Temp	2.45	2	22.91	0.11
		Gender	2.49	1	33.20	0.12
		CP x Temp	0.40	2	21.39	0.67
		CP x Gender	0.95	1	33.08	0.34
		Temp x Gender	1.30	2	33.04	0.29
		CP x Temp x Gender	1.34	2	33.83	0.27
Pr	107	**CP**	8.61	1	34.09	**<0.01****
		Temp	0.76	2	34.02	0.47
		**Gender**	5.55	1	89.52	**0.02***
		CP x Temp	0.54	2	33.97	0.59
		CP x Gender	0.61	1	90.27	0.44
		Temp x Gender	0.60	2	89.90	0.55
		CP x Temp x Gender	1.45	2	90.06	0.24

Pr: *Pieris rapae*; Pc: *Pieris canidia*

CP: Competition type (intra- vs. interspecific); Temp: Temperature.

N: sample size; *F*: ANOVA F test statistics; DF: degree of freedom; DF.res: residual degree of freedom, estimated using Kenward-Roger method; *P*: p-value, with significant terms in bold. * $P<0.05$; ** $P<0.01$; *** $P<0.001$

Supplement 6. *Post hoc* pairwise comparisons of *Pieris* traits across temperature treatment groups (18.5 ºC, 21.5 ºC, and 24.5 ºC, representing control, 3 ºC, and 6 ºC warming, respectively). Different letters indicate significant difference among temperature treatment groups at the 95% confidence level. Gray highlights indicate the cases where temperature effect differed between host plants (i.e., host plant dependent effect of warming).

**Host plant**	*Rorippa indica*				*Brassica oleracea*			
***Pieris* species**	*P. canidia*		*P. rapae*		*P. canidia*		*P. rapae*	
**Competition type**	Intra	Inter	Intra	Inter	Intra	Inter	Intra	Inter
Larval period	a-b-c	a-b-c	a-b-c	a-b-c	a-b-c	a-b-c	a-b-c	a-b-c
Pupal fresh weight	a-b-b	a-a-a	a-ab-b	a-ab-b	a-a-a	a-ab-b	a-a-a	a-a-a
Adult dry weight	a-b-b	a-a-a	a-ab-b	a-ab-b	a-a-a	a-a-a	a-a-a	a-a-a
Forewing length	a-b-b	a-ab-b	a-ab-b	a-ab-b	ab-a-b	a-a-a	a-a-a	a-a-a

Supplement 7. Pairwise comparisons of *Pieris* performance between competition types (intra- vs. interspecific competition) at each temperature, using lsmeans for the larval period (day), pupal fresh weight (g), adult dry weight (g), and forewing length (mm), and permutation tests for RGRs (ratio data; 5000 iterations). Significant P-values ($P < 0.05$) are in bold and indicate that *Pieris* from intra- and interspecific competition performed differently. Visualized results are available in Fig. 2 and 3.

Host plant: *Rorippa indica*

***P. canidia* at 18.5 $^{\circ}$C (intra- vs. interspecific competition)**

Larval period: estimate ± SE = 3.103 ± 1.015; t = 3.058, **P = 0.004**

Pupal fresh weight: estimate ± SE = -0.035 ± 0.015; t = -2.395, **P = 0.023**

Adult dry weight: estimate ± SE = -0.008 ± 0.002; t = -3.011, **P = 0.005**

Forewing length: estimate ± SE = -2.157 ± 0.912; t = -2.365, **P = 0.024**

RGR: permutation test, **P = 0.014**

***P. canidia* at 21.5 $^{\circ}$C (intra- vs. interspecific competition)**

Larval period: estimate ± SE = 0.695 ± 1.019; t = 0.682, P = 0.500

Pupal fresh weight: estimate ± SE = -0.011 ± 0.015; t = -0.765, P = 0.450

Adult dry weight: estimate ± SE = -0.003 ± 0.003; t = -1.082, P = 0.287

Forewing length: estimate ± SE = -0.619 ± 0.917; t = -0.676, P = 0.504

RGR: permutation test, P = 0.614

***P. canidia* at 24.5 $^{\circ}$C (intra- vs. interspecific competition)**

Larval period: estimate ± SE = 0.056 ± 1.001; t = 0.056, P = 0.956

Pupal fresh weight: estimate ± SE = -0.004 ± 0.014; t = -0.245, P = 0.808

Adult dry weight: estimate ± SE = -0.001 ± 0.003; t = -0.544, P = 0.591

Forewing length: estimate ± SE = -0.538 ± 0.885; t = -0.608, P = 0.548

RGR: permutation test, P = 0.969

***P. rapae* at 18.5 $^{\circ}$C (intra- vs. interspecific competition)**

Larval period: estimate ± SE = -0.102 ± 0.671; t = -0.151, P = 0.881

Pupal fresh weight: estimate ± SE = 0.008 ± 0.013; t = 0.598, P = 0.555

Adult dry weight: estimate ± SE = 0.001 ± 0.003; t = 0.430, P = 0.671

Forewing length: estimate ± SE = 0.805 ± 0.894; t = 0.899, P = 0.378

RGR: permutation test, P = 0.654

***P. rapae* at 21.5 ºC (intra- vs. interspecific competition)**

Larval period: estimate ± SE = -0.110 ± 0.671; t = -0.151, P = 0.881

Pupal fresh weight: estimate ± SE = -0.002 ± 0.012; t = -0.141, P = 0.889

Adult dry weight: estimate ± SE = -0.000 ± 0.003; t = -0.003, P = 0.998

Forewing length: estimate ± SE = -0.129 ± 0.878; t = -0.147, P = 0.884

RGR: permutation test, P = 0.880

***P. rapae* at 24.5 ºC (intra- vs. interspecific competition)**

Larval period: estimate ± SE = 0.104 ± 0.635; t = 0.163, P = 0.872

Pupal fresh weight: estimate ± SE = 0.002 ± 0.012; t = 0.151, P = 0.881

Adult dry weight: estimate ± SE = -0.0000 ± 0.003; t = -0.036, P = 0.972

Forewing length: estimate ± SE = -0.150 ± 0.847; t = -0.176, P = 0.862

RGR: permutation test, P = 0.700

Host plant: *Brassica oleracea*

***P. canidia* at 18.5 ºC (intra- vs. interspecific competition)**

Larval period: estimate ± SE = -0.492 ± 0.610; t = -0.805, P = 0.433

Pupal fresh weight: estimate ± SE = -0.024 ± 0.012; t = -1.983, P = 0.057

Adult dry weight: estimate ± SE = -0.002 ± 0.002; t = -0.945, P = 0.355

Forewing length: estimate ± SE = -0.620 ± 0.904; t = -0.685, P = 0.498

RGR: permutation test, P = 0.590

***P. canidia* at 21.5 ºC (intra- vs. interspecific competition)**

Larval period: estimate ± SE = 0.840 ± 0.602; t = 1.396, P = 0.181

Pupal fresh weight: estimate ± SE = 0.005 ± 0.012; t = 0.393, P = 0.698

Adult dry weight: estimate ± SE = 0.002 ± 0.002; t = 0.868, P = 0.393

Forewing length: estimate ± SE = 0.255 ± 0.760; t = 0.336, P = 0.739

RGR: permutation test, P = 0.831

***P. canidia* at 24.5 ºC (intra- vs. interspecific competition)**

Larval period: estimate ± SE = -0.224 ± 0.463; t = -0.483, P = 0.641

Pupal fresh weight: estimate ± SE = 0.010 ± 0.009; t = 1.106, P = 0.283
Adult dry weight: estimate ± SE = 0.0002 ± 0.002; t = 0.000, P = 0.999
Forewing length: estimate ± SE = -0.545 ± 0.565; t = -0.964, P = 0.342
RGR: permutation test, P = 0.133

P. rapae **at 18.5 ºC (intra- vs. interspecific competition)**

Larval period: estimate ± SE = 1.307 ± 0.571; t = 2.289, **P = 0.028**
Pupal fresh weight: estimate ± SE = -0.010 ± 0.010; t = -1.098, P = 0.279
Adult dry weight: estimate ± SE = -0.002 ± 0.002; t = -1.152, P = 0.256
Forewing length: estimate ± SE = -1.069 ± 0.437; t = -2.448, **P = 0.018**
RGR: permutation test, **P = 0.017**

P. rapae **at 21.5 ºC (intra- vs. interspecific competition)**

Larval period: estimate ± SE = 1.092 ± 0.541; t = 2.017, P = 0.052
Pupal fresh weight: estimate ± SE = -0.006 ± 0.009; t = -0.626, P = 0.535
Adult dry weight: estimate ± SE = -0.002 ± 0.001; t = -1.063, P = 0.295
Forewing length: estimate ± SE = -0.461 ± 0.412; t = -1.118, P = 0.270
RGR: permutation test, **P = 0.015**

P. rapae **at 24.5 ºC (intra- vs. interspecific competition)**

Larval period: estimate ± SE = 0.230 ± 0.548; t = 0.419, P = 0.678
Pupal fresh weight: estimate ± SE = -0.015 ± 0.009; t = -1.600, P = 0.119
Adult dry weight: estimate ± SE = -0.001 ± 0.002; t = -0.941, P = 0.353
Forewing length: estimate ± SE = -0.621 ± 0.418; t = -1.487, P = 0.145
RGR: permutation test, P = 0.399

Supplement 8. Potential caveats of this study.

This study raised herbivores and host plants together under warming, thereby constituting more complete warming manipulation than that of other studies that have manipulated herbivore temperature only (i.e., omitting the warming impact on the food (host plant) of herbivores). However, this approach impeded us from analyzing detailed mechanisms such as how warming might directly and indirectly affect herbivores (e.g., the warming impact on herbivore physiology and host plant quality, respectively). A more complex experimental design to test the warming impact on herbivores alone, host plants alone, and both together (direct, indirect, and combined effects, respectively) could help unearth the underlying mechanisms of the warming impact on plant-herbivore interactions. In addition, the present study was conducted under constant temperatures because of logistical concerns. Given that temperature fluctuation could potentially amplify species responses to climate warming[1], future studies with temperature fluctuation treatment (e.g., hourly) could verify our findings and add a new dimension to the marriage of species invasion ecology and climate change ecology.

Literature cited for Supplement 8

1. Paaijmans, K. P., Heinig, R. L., Seliga, R. A., Blanford, J. I., Blanford, S., Murdock, C. C. & Thomas, M. B. Temperature variation makes ectotherms more sensitive to climate change. *Global Change Biol* **19**: 2373-2380 (2013).
